# Egg producer attitudes and expectations regarding the transition to cage-free production: a mixed-methods approach

**DOI:** 10.1016/j.psj.2023.103058

**Published:** 2023-08-22

**Authors:** Vincenzina Caputo, Aaron J. Staples, Glynn T. Tonsor, Jayson L. Lusk

**Affiliations:** ⁎ Department of Agricultural, Food, and Resource Economics, Michigan State University, East Lansing, MI 48824, USA; † Department of Agricultural Economics, Kansas State University, Manhattan, KS 66506, USA; ‡ Division of Agricultural Sciences and Natural Resources, Oklahoma State University, 139 Agriculture HallStillwater, OK 74078, USA

**Keywords:** cage-free, eggs, retailer pledges, legislative deadlines, production

## Abstract

Several cage-free egg mandates and retailer pledge deadlines are set to take effect in January 2026. Yet it is unknown whether producers can transition to cage-free production at a rate commensurate with these goals. This study uses qualitative and quantitative data from 2 U.S. egg producer surveys to evaluate the operational activities of conventional and cage-free facilities, identify market challenges, and assess the expected transition timeline. Across both studies, producers indicated higher fixed and variable costs in cage-free housing systems, with capital and labor being 2 of the central drivers of the cost increase. While several producers are willing to adopt cage-free production, they are hesitant to view the market shift as an opportunity. Among the most commonly cited barriers are limited customer demand, high capital costs, and a contradiction to environmental sustainability and food security efforts. With the current challenges, respondents are skeptical that the industry will meet the January 2026 voluntary pledge deadlines. The results from this study offer a holistic view of the potential ramifications of the cage-free transition on the egg market and can be used to inform marketing strategies and policy discussions.

## INTRODUCTION

The United States is the second largest egg producer in the world, with 111 billion eggs produced in 2021 (USDA NASS, 2022a). Of the 329 million laying hens, 29.3% are housed in cage-free systems (UEP, 2023b). This share has steadily increased over the past decade due to changes in cage-free egg state regulations, retailer pledges, and final consumer demand. To date, 10 states have passed legislation to transition to 100% cage-free eggs (Ufer, 2022), and many of the largest U.S. retailers (e.g., Kroger, Meijer, Wal-Mart) have pledged to go entirely cage-free by January 2026. According to Lusk (2019), approximately 75% of egg facilities must be cage-free to meet these goals. Yet, given current market conditions and barriers across the supply chain, it is unknown whether egg producers can transition at a rate proportionate to retailers’ cage-free pledges. With these looming deadlines, it is critical to assess the impact of this shift on various actors across the U.S. egg supply chain.

This paper uses data from 2 producer studies to assess the differences in conventional and cage-free production, identify the dynamics underlying the cage-free transition, and understand the challenges, opportunities, and potential unintended consequences of this shift. The first study uses semistructured qualitative interviews with egg producers, while the second uses quantitative survey data to obtain more comprehensive feedback. Both studies centered on 4 themes, including a general overview of business operations, a comparison of production systems (cage-free vs. conventional), an assessment of current and future business plans, and an outlook on the perceived future of the industry. Collecting and analyzing these interview and survey data enables a thorough evaluation of the impending transition through the unique lens of the producer.

Few studies have assessed the attitudes, preferences, and decision-making processes of producers in response to food policy shifts. Instead, much of the literature focuses on the consumer side of the market, evaluating consumer willingness to pay for animal welfare attributes (Allender and Richards, 2010; Chang et al., 2010; Ortega and Wolf, 2018; Lusk, 2019) and the vote-buy gap of stated policy preferences and revealed purchasing behavior (Norwood et al., 2019; Paul et al., 2019). Other studies have evaluated the welfare effects of cage-free legislation and their anticipated market effects (Malone and Lusk, 2016; Mullally and Lusk, 2018; Carter et al., 2021; Hopkins et al., 2022). For example, Oh and Vukina (2021) predict that California's ban on conventional eggs will result in an annual welfare loss for California consumers and retailers of $72 million and $23 million, respectively.

Past studies focusing on producer decision-making and expectations following animal welfare policy shifts include Tuyttens et al. (2011) and Stadig et al. (2016). Both studies focus on Belgian egg producer sentiment in response to the European Union (**EU**) ban on conventional cages starting on January 1, 2012 (EU Directive 1999/74, 1999). Tuyttens et al. (2011) focus on producer expectations after the ban was announced but 2 yr before the policy was implemented. Their results suggest that the average producer believes cage-free systems are inferior to conventional systems in labor requirements, farm profitability, and hen health. Stadig et al. (2016) followed up with these egg producers in 2014, providing evidence of higher fixed and variable costs, decreased farm profitability, and farmers exiting the market due to the stricter production guidelines.

In understanding how the results from these studies may translate to the larger U.S. market, it is important to consider how the EU and U.S. egg markets differ. Most notably, the EU market is much less consolidated, with more firms operating smaller production facilities. Indeed, the largest producer in Tuyttens et al. (2011) had 181,000 laying hens. In the United States, the largest 40 egg producers have more than 1 million laying hens, with the top 5 having more than 10 million (WattPoultry, 2022). Further, Belgian egg production accounts for just over 2% of EU production (Windhorst, 2017). Thus, while some of the findings from these studies may translate to the U.S. egg market, it is critical to understand the expected supply chain ramifications of the transition to cage-free. This is particularly true given that much of the transition hinges on the voluntary commitment to pledges rather than formal government regulations.

This study offers 3 primary contributions. First, the study compares conventional and cage-free production systems, discussing the cost structure of different hen-housing systems. Producers describe conventional as superior to cage-free in food affordability, production efficiency, and environmental sustainability goals. Capital and labor costs are the 2 expenses with the largest cost differential between systems. Second, we outline the primary barriers to cage-free adoption and describe the potential unintended consequences of the transition. Limited customer (retailer) demand, high capital costs, and tradeoffs with environmental sustainability and food security goals are the most commonly cited barriers, while industry consolidation is expected among producers. Finally, summarizing the results of the 2 studies, we describe the expected timeline for the cage-free transition and offer discussion on the implications across the wider supply chain to help inform marketing strategies and policy discussion.

The remainder of this paper is structured as follows. “Study 1: Semistructured Qualitative Interviews” section summarizes the methods and results of the qualitative study, while “Study 2: Quantitative Survey Results” section describes the quantitative survey. “Discussion and Implications” section connects the main findings across the 2 studies and discusses the market and policy implications. “Conclusions” section concludes.

## STUDY 1: SEMISTRUCTURED QUALITATIVE INTERVIEWS

### Methods and Procedures

In June 2022, we held 7 semistructured individual interviews with U.S. egg producers via Zoom.^1^ Interviewees were selected based on recommendations from the President & CEO of the United Egg Producers (**UEP**), the largest national Capper-Volstead cooperative of egg producers representing approximately 92% of the U.S. egg industry (UEP, 2023a). Each interview lasted between 45 and 75 min. Interview questions were developed through previous conversations with industry stakeholders and arranged into 4 themes: i) current business operations, ii) comparison of production systems, iii) business plan and relationship with buyers, and iv) future industry directions. Table 1 presents the thematic areas and core questions, while the questionnaire is available as supplemental material. Probing questions were also asked to explore issues in more detail, while closing questions were used to gain additional egg production-related information or answer producer questions.

**Table 1:** Thematic areas and core questions in the qualitative interviews with producers.

Thematic areas	Core questions
Business profile	• Can you please provide a brief overview of your current production setup? This could include your annual production, geographic location(s), variety and type of egg products, etc. • What share of your egg production is from cage-free facilities?
Comparing conventional and cage-free production	• How has the switch to cage-free affected your labor demand, capital requirements, feeding process, safety and quality control, and disease management?
Business plan and relations with buyers	• What percentage of your eggs do you sell under contracts, and how long have you had these relationships with your buyers? • How often do you communicate with your buyer about the transition to cage-free eggs? • Does your company have an annual business plan for the transition to cage-free? Does this plan include strategies on what to do with the outsourced cages? • How difficult is it to finance the new construction of cage-free facilities, and have there been significant barriers to capital?
Future industry directions	• How do you perceive the legislative changes occurring in different states toward going 100% cage-free? • What do you perceive as the biggest challenges to converting to cage-free?

Individual responses were then transcribed, and a qualitative content analysis approach (Patton, 2002) was used to map the text data to one of the 4 survey themes: i) current business operations, ii) comparison of production systems, iii) business plan and relationship with buyers, and iv) future industry directions. For example, any transcribed producer response data discussing barriers or opportunities to further adoption were placed in the fourth theme (future industry directions). Patterns and consistencies across each theme were identified by comparing across participants and were summarized to gauge general producer expectations and sentiment.

### Results

#### Producer Profile

Table 2 highlights the producer profiles from the 7 qualitative interviews. Annual production, employment, and precise cage-free adoption rates are withheld to maintain producer anonymity. Collectively, these producers represent more than 25% of the U.S. egg industry, cover shell egg and liquid egg marketplaces, and supply retailers and food manufacturers. Six producers had less than 30% cage-free production, while one had more than 60%.

**Table 2:** Egg producer profiles.

Producer	Farm size	% Cage-free	Customers	Egg types
A	Small	<30%	Grocery stores	Shell egg
B	Medium	<30%	Food service companies	Liquid eggs
C	Large	<30%	Grocery stores & food service companies	Liquid eggs & shell eggs
D	Large	<30%	Grocery stores	Shell eggs
E	Large	<30%	Grocery stores & food service companies	Liquid eggs & shell eggs
F	Large	<30%	Food service companies	Liquid eggs
G	Large	>60%	Grocery stores & food service companies	Liquid eggs & shell eggs

#### Comparing Operational Activities

**Figure 1:**
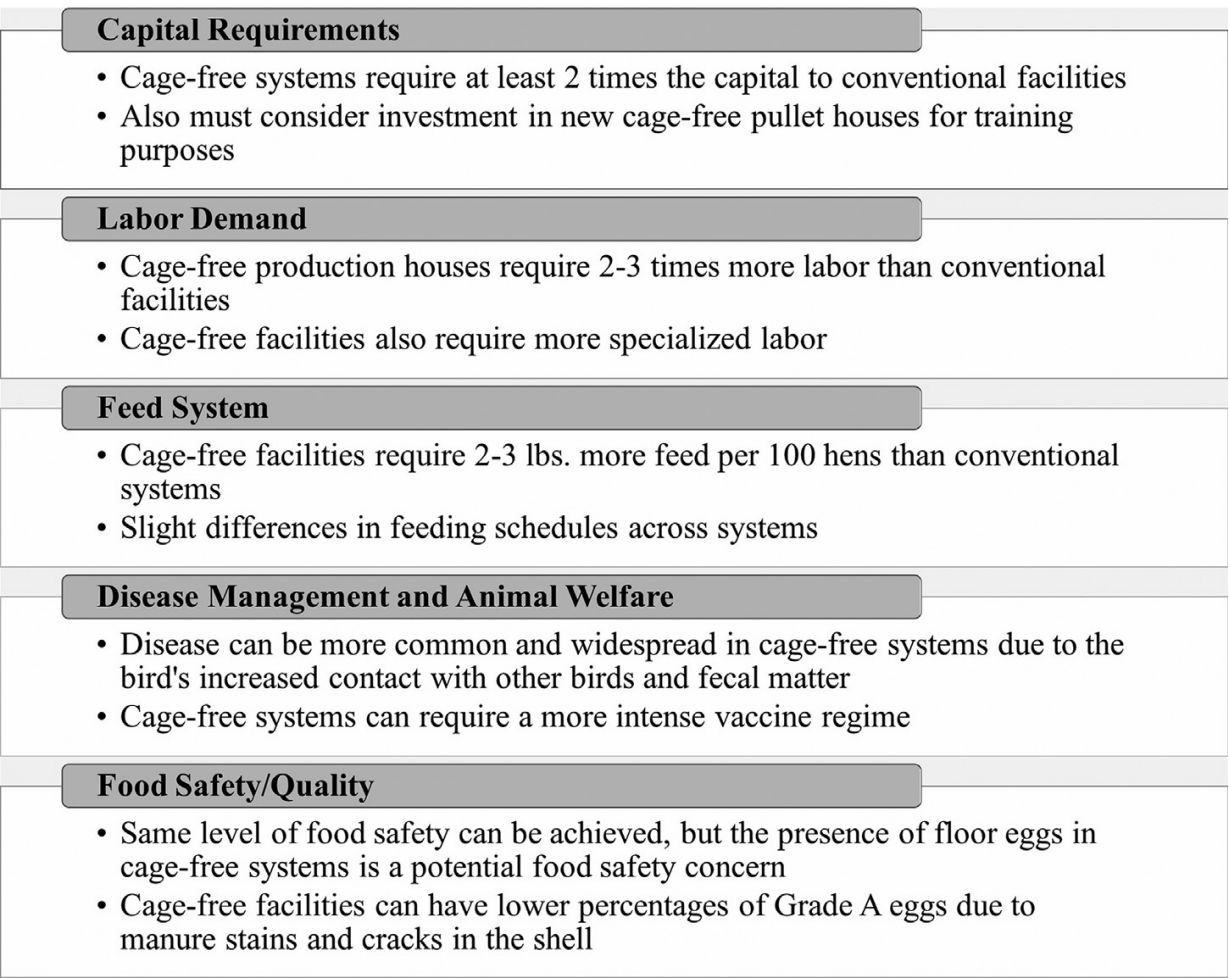
Cage-free system operational activities discussed with producers.

Respondents were asked to compare and contrast the operational activities of cage-free and conventional systems to better understand the cost and production differences. Specifically, interviewees were asked to describe how cage-free production affects 5 factors: i) labor demand, ii) capital requirements (infrastructure), iii) feed system and associated costs, iv) disease management, and v) food safety/quality. Figure 1 summarizes the results.

All 7 producers stated that cage-free systems require at least 2 times the capital of conventional facilities, and 6 recommend building greenfield facilities when possible. Producers also suggested a necessary investment in cage-free pullet houses, describing the importance of the bird spending its early life in a cage-free environment for proper training. For instance, Producer A stated, “Try to get that pullet as well-trained in that [cage-free] equipment, so they know where to lay the eggs, they know how to go up and down the system to get feed and water. Once you've lost that training period, you're never going to get that back.”

The second factor that producers unanimously discussed was the increased labor demand in cage-free systems. Speaking of their own experiences, producers reported that labor demand in cage-free systems could be 2 to 3 times as high as that of conventional production. Additionally, Producer C stated that cage-free environments require “much more of an animal care role where people are actually interacting with the birds, and they have to be much more attuned to the behaviors. It requires a lot more [worker] training, a lot more experience, and a lot more care… [and] also a different labor rate.” Given the more specialized tasks and the enhanced need of fulfilling a “more animal husbandry role” (Producer D), the pool of potential employees shrinks, and the wage rate likely increases.

In addition, businesses will incur added costs due to an increased need for feed, where Producers E and F suggest approximately 2 pounds more feed per 100 birds in cage-free systems. Assuming 22.5 pounds per 100 lbs in a conventional system (Producer F), these estimates suggest a 9% increase in feed intake for cage-free flocks relative to conventional ones.^2^

The increased contact of the birds with feces and other birds in a cage-free environment increases the disease risk, requiring a more intensive vaccine regime (Producer E) and heightened disease testing requirements (Producer C). The disease pressure and rigorous vaccine regime present long-term risks and costs to producers. This concern is becoming increasingly salient, as record-breaking avian influenza cases have forced U.S. poultry producers to cull more than 58 million birds in 2022 (CDC, 2023), contributing to record-setting egg prices (BLS, 2023).

Mortality rates across production systems, however, were a more debated topic among the producers. Three (Producers B, D, and E) suggested that mortality rates can be greater in cage-free systems due to the birds’ interaction with their fecal material, more cannibalistic behavior, and the birds’ protective instinct to “crowd” one another. However, Producer C stated that mortality rates were similar across systems but agreed it could be higher in the first few years as the producer adjusts to the transition.^3^

One concern with cage-free production not present in conventional systems is floor eggs (i.e., any egg laid on the litter floor). Floor eggs are a food safety concern, as it is difficult for workers to know how long the egg has been on the floor, and there is a higher probability of disease contamination.^4^ There is also a food quality concern from floor eggs. Producer E reported that 94 to 97% of conventional eggs are Grade A, while cage-free typically ranges from 88 to 94%. According to Producer D, the difference in grading stems from more frequent manure stains and shell cracks on cage-free eggs.

#### Business Plan and Relations With Buyers

Producers provided a broad overview of their contractual obligations, including an estimated number of buyers and the percentage of eggs for shell-egg and liquid-egg markets. Each producer sells most of their eggs through formal contracts to a few primary customers.^5^ Most of these contracts were long-lasting relationships, with some spanning multiple decades. Spot markets comprise a small share of annual sales, ranging from 0 to 20% for our 7 producers.

Comparing customer bases with cage-free adoption rates, there appears to be an important distinction between the shell-egg and liquid-egg marketplaces. For example, consider the perspective of Producer B, who supplies all liquid eggs:

“We're not marketing to retailers and consumers who are looking at an egg as an egg. We're marketing to big branded processed food companies where we are 1 of 5 to 10 ingredients on a back panel. The consumer is not as locked in on the choice of am I eating an egg, and where does it come from? They just want to make pancakes.”

This suggests that the cage-free attribute is more salient on a carton of eggs than on a processed food box while also indicating a difference in how consumers think about eggs as a final product vs. eggs as an ingredient.

While 3 out of 7 producers had ongoing cage-free projects, these were always customer driven. Each producer stated they would not engage in speculative building based on the voluntary and informal pledges made by retailers, and they were not interested in federal support because subsidies would not solve the long-term issue of finding a buyer. Thus, producers will only transition at the request of their customers, driven by the need to demonstrate a long-term buyer to obtain funding from a bank. Without proof of a long-term commitment from the customer, banks may be hesitant to approve the loan given the significant capital investment.

According to some producers, if mandates restricting conventional production are imposed, all producers will be forced to transition at once, and obtaining funding could become more difficult. This is particularly true because as cages lose value, producers may be unable to claim them as collateral, increasing the producer's debt-to-asset ratio and making a bank less likely to lend. Smaller producers are more likely to be negatively affected by this precautionary lending.

#### Perceptions of the Industry's Future

Producers were asked what, in their perspective, were the (3) largest barriers and (3) opportunities to expand cage-free production. Some producers struggled to provide a complete list of opportunities, while others were skeptical of viewing the transition as an opportunity. Producer A, for example, stated that the transition is “more of a risk than an opportunity because of the capital requirements and the unknowns.” Producer B suggested limited opportunities in the liquid egg marketplace but noted, “If everybody's operating under the same rules… I think the industry can become more efficient. The more volume and scale people have in the arena, the better they are going to get at it.”

**Figure 2:**
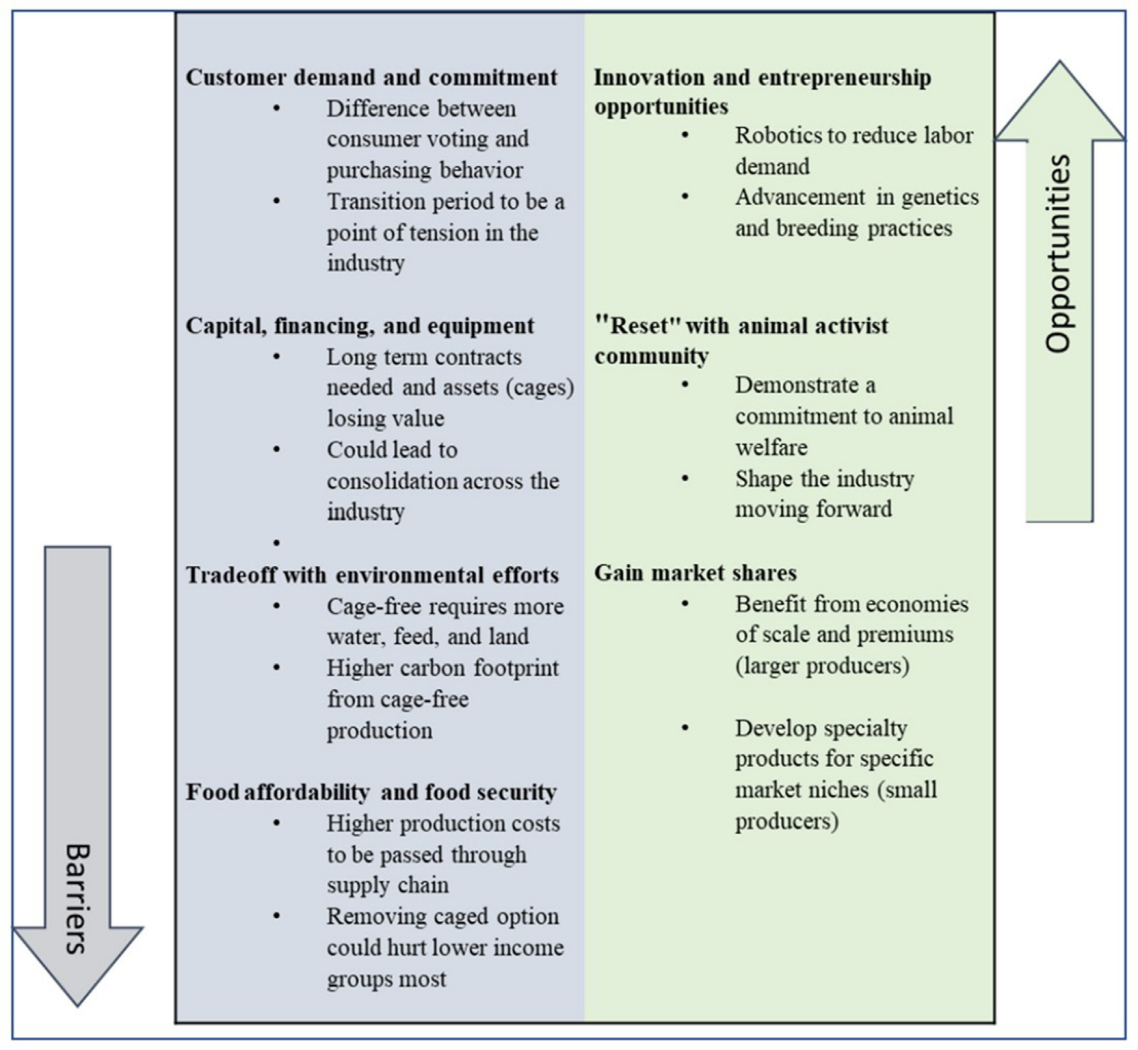
Main barriers and opportunities to cage-free adoption mentioned by producers.

Figure 2 summarizes the most commonly cited barriers and opportunities to cage-free adoption. The primary challenges include limited customer demand, capital financing, environmental sustainability, and food security. Potential opportunities include innovation and entrepreneurship, an industry “reset” with the animal activist community, and gaining market share. Each of these barriers and opportunities is discussed in greater detail below.

##### Barriers

**Barrier 1—Customer demand and commitment.** Customer demand and commitment was the primary concern raised by each producer during the interview. Summarizing this concern, Producer E stated, “Retailers that did try to make the decision when the conventional option was still present saw consumers continue to buy the conventional option. Then, when they subsequently removed the conventional option, they experienced a significant decline in egg sales.” This response addresses 2 key issues. First, retailers will find it difficult to attain cage-free goals when consumers still have the option to purchase cage eggs. There is a disconnect between the consumers’ stated production preferences and revealed purchasing behavior (Norwood et al., 2019; Paul et al., 2019). Retailers may assume that consumers prefer cage-free given the recent voting trends and stated preferences for stricter production practices. Yet, when it comes time to purchase eggs, consumers continue to buy the cheaper, conventional option when available.^6^ Second, Producer E's quote demonstrates that no retailer has a first-mover advantage in states without a cage-free mandate. If a retailer voluntarily removes the conventional alternative from their shelves, they may experience a decline in egg sales as consumers shop at a competitor's store for the cheaper option. Instead, there appears to be an agreed-upon, soft commitment to a January 2026 deadline for all the major retailers to go cage-free.

The vote-buy gap and soft commitments create uncertainty and risk in the supply chain, where different actors have different incentive structures. Retailers want producers to invest in cage-free facilities to be ready for the hard switch, but retailers are hesitant to increase cage-free purchases during the transition given the current consumer demand. Producers, on the other hand, will only invest in cage-free once they have a long-term commitment from their customers, which implies an additional 2 to 3 yr on the transition timeline for constructing these cage-free facilities. As such, the transition period is likely to be a point of tension within the industry.

**Barrier 2—Capital, financing, and equipment.** When asked about the 3 limiting factors to cage-free production, Producer F's response was “Capital, capital, and capital.” Producers, as a whole, agreed that cage-free capital requirements are at least double that of conventional systems, and long-term contracts are required before requesting funding from a bank. While securing financing has not yet been an issue for these producers, Producers A and D acknowledged it could become a concern if producers were forced to transition simultaneously.

Even with a long-term commitment and bank approval, 4 producers (Producers C, D, E, and F) stated that building cage-free facilities takes approximately 2 to 3 yr. Moreover, permitting concerns and the construction of cage-free pullet houses alongside the laying facilities can add to this timeline (Producers D and F). Limited equipment suppliers and ongoing supply chain constraints could present additional hurdles and delay the speed at which the industry can convert to cage-free production (Producers C, D, and E).

**Barrier 3—Environmental sustainability efforts.** Producers were concerned that consumers, policymakers, and actors across the supply chain do not understand the tradeoff between cage-free and environmental sustainability (Producers B, C, D, and E). According to at least 4 of the 7 producers, cage-free production requires more water, feed, and land while also having a higher carbon footprint. Thus, in these producers’ perspective, policymakers and consumers must have a holistic view of the production system before proceeding with the transition. To this point, Producer D stated, “It's hard to scale it back when it's time to say, all right, we got to get back to the environment. So, I really hope we don't make too many wrong turns that are hard to undo if it does become apparent soon that the environment is what we really need to be focused on.”

**Barrier 4—Food affordability and security.** Inflation and other supply chain disruptions are driving up food prices and heightening concerns over food affordability and insecurity (Ahn and Norwood, 2021; Gundersen et al., 2021). Four producers (Producers B, D, E, and F) explicitly mentioned the conversion to 100% cage-free as a food security issue. The higher production costs imposed by more restrictive animal production policies are typically passed down the value chain and borne by the consumer (Mullally and Lusk, 2018).

##### Opportunities

**Opportunity 1—Avenues for innovation and entrepreneurship.** For opportunities, producers state potential for innovation and entrepreneurship in the support functions and processing side of the supply chain (Producers B and C).^7^ Producers C and D emphasized the need for advancements in robotics to help address the labor challenge. Producers D, F, and G also mentioned advances in ventilation and other technologies to create a more sanitary environment and dry the manure. For example, Producer G has installed in-floor heating to dry the manure so the robots can sweep it; it also reduces the smell for workers. Relatedly, Producers F and G discussed ways to convert manure into fertilizer, turning a byproduct of production into a new product line.

**Opportunity 2—“Reset” with the animal activist community.** Over the past few decades, there has been an increase in consumer demand for animal welfare attributes in food (Lagerkvist and Hess, 2011; Clark et al., 2017). At the same time, there has been an increase in corporate responsibility for animal welfare standards (Kapelko and Oude Lansink, 2022). Producer C stated that the conversion to cage-free represents an opportunity to phase out older production systems and improve relationships with the animal activist community. One example of this commitment is Producer G's zero-tolerance policy for poor bird management. Other avenues include modeling future facilities to incorporate animal-friendly technologies such as recovery pens (Producers C and G). As producers become more experienced with these systems, technological and production advancements may also improve efficiency and animal welfare outcomes (e.g., declining mortality rates in cage-free systems, as noted by Producer A).

**Opportunity 3—Gain market share.** Whether cage-free is viewed as an opportunity may depend on a company's size. In general, larger producers benefit from economies of scale and have larger customers, making funding easier to acquire. Small producers, however, may find it challenging to compete in this space due to high capital costs and barriers to entry. For example, Producer F noted, “I'm afraid that the industry is going to continue to consolidate… There are a lot of producers out there that are struggling to figure out what to do. Do we invest in cage-free? Do we sell the business? Do we let somebody else worry about it? I think those are all valid questions that every producer in the egg business is trying to figure out and are struggling with today.” Larger producers could view the transition period as an ideal time to acquire competitors and gain market share.^8^

## STUDY 2: QUANTITATIVE SURVEY RESULTS

### Methods and Procedures

Following the first study, we developed a quantitative survey to distribute to a broader group of egg producers, and data were collected from September to November 2022. The UEP President & CEO distributed an email containing a Qualtrics survey link and encouraged all UEP members to complete a survey. In total, 29 U.S. egg producers completed the survey.

The survey was built using the 4 main themes presented in the qualitative study, and additional questions were added for a more comprehensive and statistical overview of the market.^9^ Table 3 summarizes the key concepts and central questions from the quantitative study. The Appendix accompanying this manuscript contains a copy of the survey instrument used in this study.

**Table 3:** Thematic areas and core questions in the quantitative survey of producers.

Thematic areas	Core questions
Business profile	• What share of laying hens in your operation are currently housed in cage-free systems? • What share of the eggs you currently produce is sold through each of the following outlets (must sum to 100%)? Including retail, food service, manufacturing, and exports. • What description best aligns with the current size of your combined laying hen operation?
Comparing conventional and cage-free production	• Which production method (conventional or cage-free) ranks highest, or best, in the following categories? Including sustainability, animal welfare, production efficiency, food affordability, and environmental impact. • What best reflects the annual percentage change in costs and revenue for your operation when operating cage-free vs. conventional? • What best reflects your expected return on investment (ROI) when operating a cage-free and conventional facility?
Business plan and relations with buyers	• Currently, what share of your conventional/cage-free eggs are sold under the following egg pricing methods (must sum to 100%)? Including i) spot markets, ii) external price, and iii) cost-plus
Future industry directions	• Looking forward to January 2026, what share of laying hens in the United States do you think will be housed in cage-free systems? • What best aligns with how you view the balance of opportunity and challenge facing the U.S. egg industry and the possible increase in cage-free production?

After asking the respondents questions about their operation (e.g., current flock size, region), they were asked to compare conventional and cage-free systems. First, they were asked to state which production system ranks best in the following metrics: sustainability, animal welfare, production efficiency, food affordability, and environmental impact. The definition of these terms was left to the producers’ discretion, which implies a degree of subjectivity in the responses. They were then asked to compare each system's cost structures, expected revenues, and ROIs to understand how the transition may affect farm profitability.

To assess their current marketing strategy, respondents were asked to state the percentage of eggs sold under various pricing arrangements. Options included spot markets, external price, and cost-plus, and the answers had to sum to 100%. Spot markets refer to the “cash” market where no contractual agreement exists. An external price contract is a formula contract where the price received is tied to the Urner Barry reported price (Urner Barry, 2023). A cost-plus contract is a formula-based contract where the price received is linked to feed or other production costs.

Lastly, in assessing the future of the industry, respondents were asked to state the share of cage-free eggs they expect to be in the market as of January 2026. Respondents could choose from a drop-down menu ranging from “0–5%” to “96–100%,” allowing us to plot a distribution to capture the diverse perspectives of the producers.

The data were exported from Qualtrics and imported into the software program Stata (StataCorp, 2023). Here, we perform our statistical analysis, including computing traditional summary statistics and tabulations, to obtain a more comprehensive assessment of producer attitudes and expectations of the transition to cage-free eggs.

### Results

#### Producer Profile

This analysis focuses on the completed responses from 29 UEP members.^10^ Consistent with the need to assure anonymity and encourage survey participation, questions on current laying hen operation size, the proportion of cage-free production, etc., were presented in ranges and are presented only in the aggregate. Table 4 provides summary statistics for the sample.

**Table 4:** Summary statistics for quantitative study (*n* = 29).

Variable	% of respondents
Production size	
Less than 500,000	0.0%
500,000–1 million	13.8%
1 million–2 million	31.0%
2 million–3 million	13.8%
3 million–4 million	3.4%
4 million–5 million	0.0%
More than 5 million	37.9%
Current percentage of cage-free eggs	
0–20%	25.0%
21–40%	39.4%
41–60%	7.1%
61–80%	7.1%
81–100%	14.3%
Avg. share of sales to different outlets	
Retail (grocery)	59.5%
Food service (restaurants)	24.3%
Manufacturing	11.1%
Export	1.6%
Region	
Midwest	48.0%
Northeast	16.0%
South	8.0%
West	28.0%
No. of years remain in business	
5 yr or less	10.3%
6–10 yr	3.4%
11–15 yr	0.0%
16–20 yr	0.0%
More than 20 yr	86.2%

For operation size, 7 ranges were presented, spanning from “Less than 500,000 laying hens” to “Over 5 million laying hens.” We use mid-point levels of each range to estimate the market share represented in the sample. It is important to note that 11 of the 29 respondents selected the largest presented size. If we first assume a conservative average value of 5.5 million for each of these respondents, then combined, the 29 respondents may represent about 90.5 million laying hens. Knowing some operations exceed 10 million laying hens (WattPoultry, 2022), this estimate is likely too low. If we alternatively assume 9.5 million applies to the 11 selecting the largest presented size range, then the 29 respondents may represent about 134.5 million laying hens. With approximately 300 million layers under UEP membership, we are confident that the 29 complete respondents represent over 30% of UEP membership production, and our best assessment is that about 50% of UEP membership production is represented.

On average, respondents indicate that 38% of their laying hens are currently housed in cage-free systems. This reveals that current cage-free adoption is higher among survey respondents than the national average (29%). However, there is wide variation in cage-free adoption rates, as 3 respondents indicate 0 to 5% cage-free production, and 4 state 96 to 100% cage-free production. There is also sufficient regional variation, where 48% of producers are from the Midwest; 28% are from the West; 16% are from the Northeast; and 8% are from the South. USDA NASS (2022b) states that the Midwest accounted for approximately half of all U.S. egg production in 2021; 27% for the South; 12% for the West; and 11% for the Northeast. Even after adjusting for production by region, the sample underrepresents production in the South and overrepresents the West. Nonetheless, this section reports simple averages (rather than weighting responses) due to an inability to confidently extrapolate the available sample data to the national level in a manner consistent with the industry's actual size distribution.

#### Comparing Operational Activities: Cage-Free Vs. Conventional

**Figure 3:**
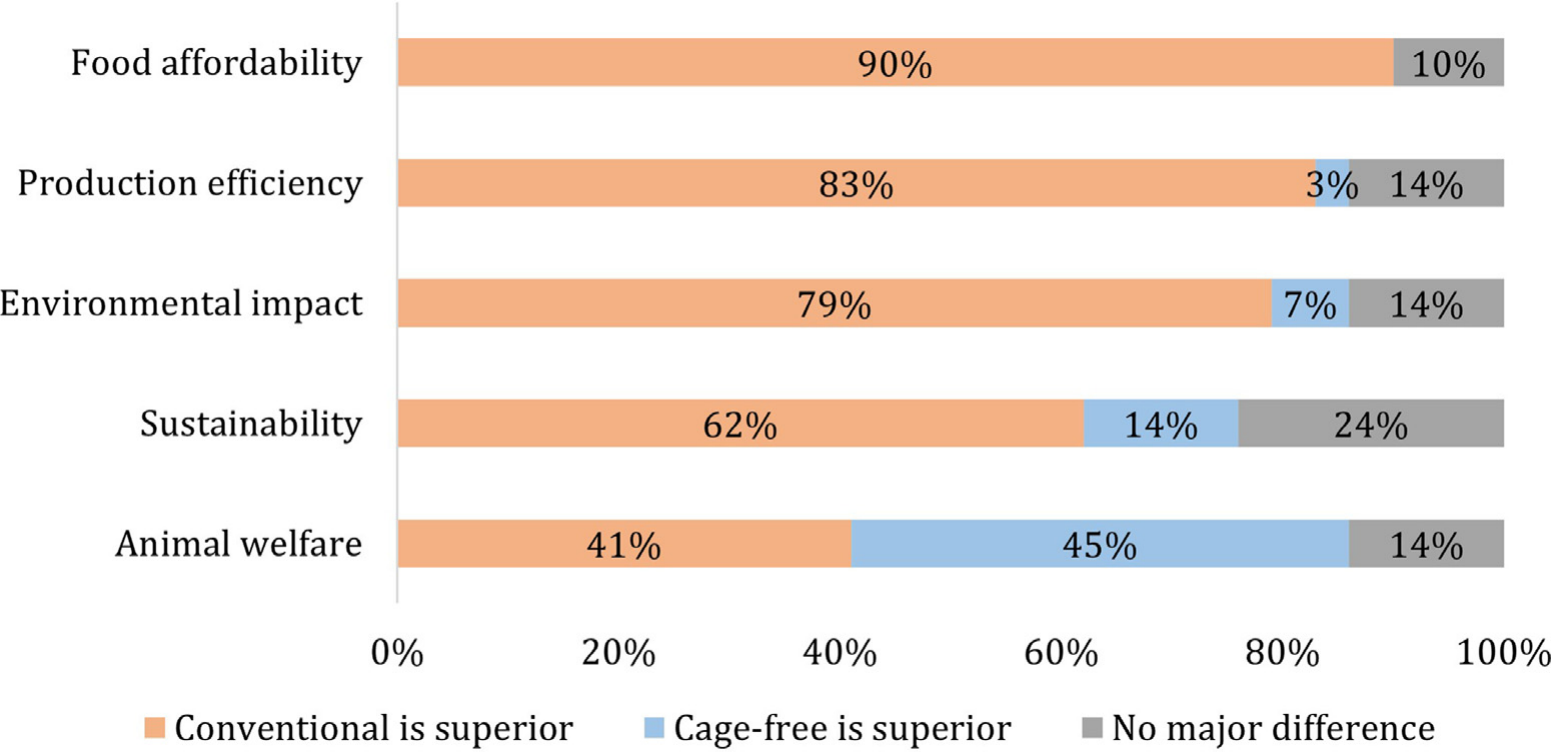
Views on which production method ranks best across different characteristics.

To gain a general understanding of sentiment toward cage-free systems, producers were asked to select the housing system that ranked best in sustainability, animal welfare, production efficiency, food affordability, and environmental impact. Figure 3 summarizes the results.

Overall, UEP members view conventional housing as superior in food affordability (90%), production efficiency (83%), and environmental impact (79%). Food affordability and environmental impact concerns align with the results of the qualitative interviews, while results to a follow-up question suggest cage-free productivity is 4% lower than conventional, on average. Specifically, respondents were asked what productivity they expected in January 2026 for each production system. The average cage-free yield is expected to be 11 eggs per yr lower, with 295 eggs per yr projected for conventional and 284 for cage-free systems. A slight majority (62%) view conventional systems as best for sustainability, with about one-fourth seeing cage-free and conventional as equivalent. Animal welfare is the only area where more producers (45%) view cage-free as superior.

**Figure 4:**
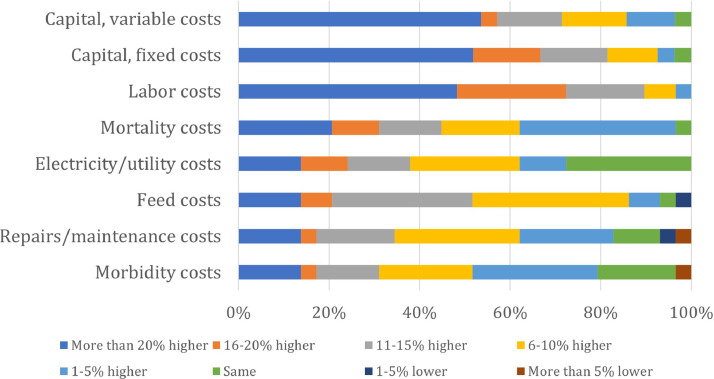
Expected change in annual costs across categories when building new cage-free facility relative to conventional.

Producers were also asked to consider how cage-free annual expenses would be impacted relative to conventional production if building a new cage-free facility, holding the number of laying hens constant. This was done across 8 categories, with responses ranging from “More than 5% less” to “More than 20% higher.” Figure 4 summarizes the results.

There are few instances where cage-free is believed to have lower expenses than conventional. In fact, the most common response for capital (fixed and variable) and labor costs was that cage-free expenses are at least 20% higher. If one presumes 25% reasonably applies to this highest presented category, then an average cost difference can be derived from the survey responses using interval-censored models. Doing so suggests cage-free expenses would be 8 to 19% higher than conventional systems.^11^ However, the estimated 8 to 19% higher costs for cage-free systems should be seen as a lower bound estimate of the anticipated cost, as the results of the qualitative interviews suggested that capital and labor can be more than double the rates of conventional.

In considering revenue, respondents were asked to consider how expected cage-free revenue would compare to conventional production, again presuming the number of laying hens remains constant. Three respondents (11%) anticipate more than 5% lower revenue from cage-free, 4 respondents (14%) expect the same revenue in cage-free and conventional systems, and the remaining respondents (75%) expect cage-free revenue to be higher. From these responses, we derive an average implied estimate that cage-free revenue will be 8% higher than conventional.^12^ Instead, using return on investment (**ROI**), the average expected ROI was +10.3% for cage-free (with a 95% confidence interval of 8.0–12.6%) and +10.6% for conventional (with a 95% confidence interval of 8.7–12.4%).

A key take-home point is worth highlighting. The 8% revenue impact is on the lower end of cost impacts. Meanwhile, a similar ROI is expected when directly assessed. While this suggests some inconsistencies, combined, these points align with ongoing innerindustry discussions around the transition in housing being complicated and varied.

#### Business Plan and Relations With Buyers

The average producer supplies 59% of eggs to retail, 24% to food service, 11% to manufacturing, and 2% to exports (Table 1). Of course, averaging across producers masks considerable variation in marketing strategies that are worth noting. For example, 5 (17%) producers state that none of their eggs are sold to retail outlets, while 3 (10%) sell solely to retailers. Projecting to January 2026, the average producer expects a decline in the share of their eggs to move through retail outlets (52% in January 2026), with slight differences expected in the other 3 outlets.

There is a positive correlation between a firm's share of cage-free production and the percentage of sales to retail outlets (0.50) and a negative correlation between cage-free production and sales to food service (−0.43) and food manufacturing (−0.30). This supports the conjecture from the qualitative study that the cage-free attribute is more salient on a carton of eggs vs. a liquid egg marketplace. Looking forward to January 2026, the correlation between the respondent's expected share of cage-free production and expected sales through retail outlets retains a similar magnitude (0.51) while the correlation with food service weakens (−0.21). Alternatively, the negative correlation between the share of cage-free production and sales through food manufacturing strengthens (−0.44). This dynamic suggests that food away from home settings (e.g., restaurants, fast food) may begin marketing cage-free eggs more frequently than food manufacturing companies.

**Figure 5:**
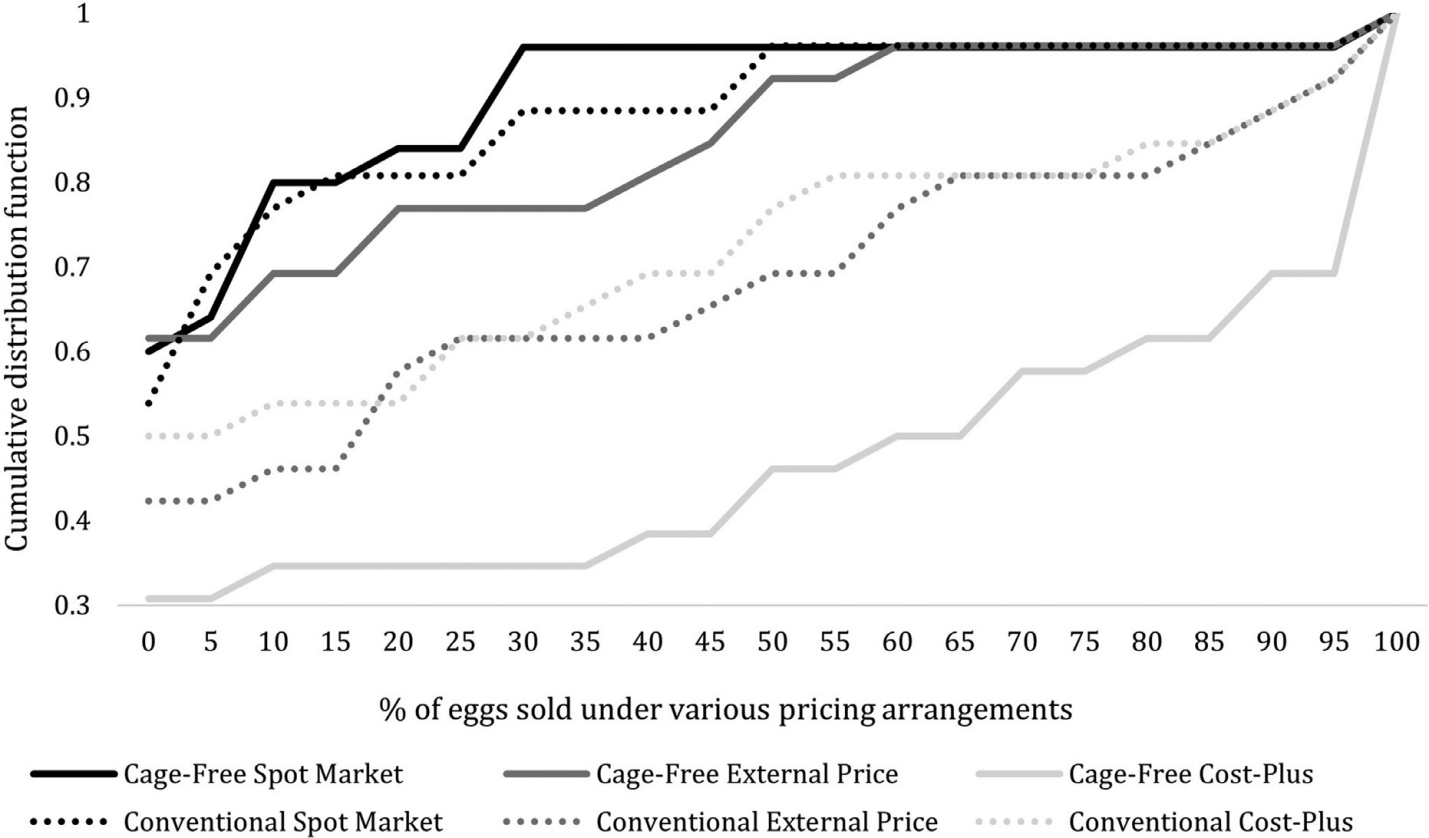
Cumulative distribution function of the percentage of eggs sold under various pricing arrangements, conventional vs. cage-free.

In assessing their contracts with their customers, respondents were asked to report the share of eggs tied to different egg pricing methods, including external price, cost-plus, and spot markets. This series of questions was repeated for conventional and cage-free eggs to explore differences in the current market structure. Figure 5 reports the distribution of responses indicating the share of each egg type tied to various pricing methods.

In the conventional egg market, 32% of conventional eggs are sold with contracts tied to an external price, 29% using cost-plus contracts, and 11% using spot markets, on average. Cage-free eggs are more likely than conventional eggs to be sold using cost-plus contracts. On average, 14% of cage-free eggs are sold with contracts tied to an external price, 49% using cost-plus contracts, and 9% of cage-free eggs are sold on the spot market. Figure 5 provides additional insights other than the means, including key differences between the conventional and cage-free marketplace. First, producers prefer to avoid spot markets, particularly in cage-free settings. Approximately 60% of producers state that they do not use spot markets for cage-free eggs, compared to 54% for conventional markets. The most notable difference, however, comes in using cost-plus contracts in the cage-free market compared to conventional settings. Approximately 38% of producers sell 90% or more of their cage-free eggs through cost-plus contracts vs. 12% for conventional eggs. The preference for cost-plus contracts may stem from the added expenses and increased variability in the cage-free system, as discussed earlier.

Respondents using contracts tied to external prices or with cost-plus considerations were presented with a follow-up question to assess the typical length of these agreements. While there was one instance of a conventional egg external price-based contract exceeding 15 yr in length, all other responses indicated durations of 5 yr or less, with 2 yr being the most common. On the cage-free side, the longest duration of external price-based contracts for cage-free eggs was 3 yr, with 2 yr being the most common. The reported duration of cost-plus based, cage-free contracts vary more. While 2 yr remained the most common duration, 3 respondents indicated contracts of 8 or more years, and 2 others reported 5-yr contracts.

#### Perceptions of the Industry's Future

**Figure 6:**
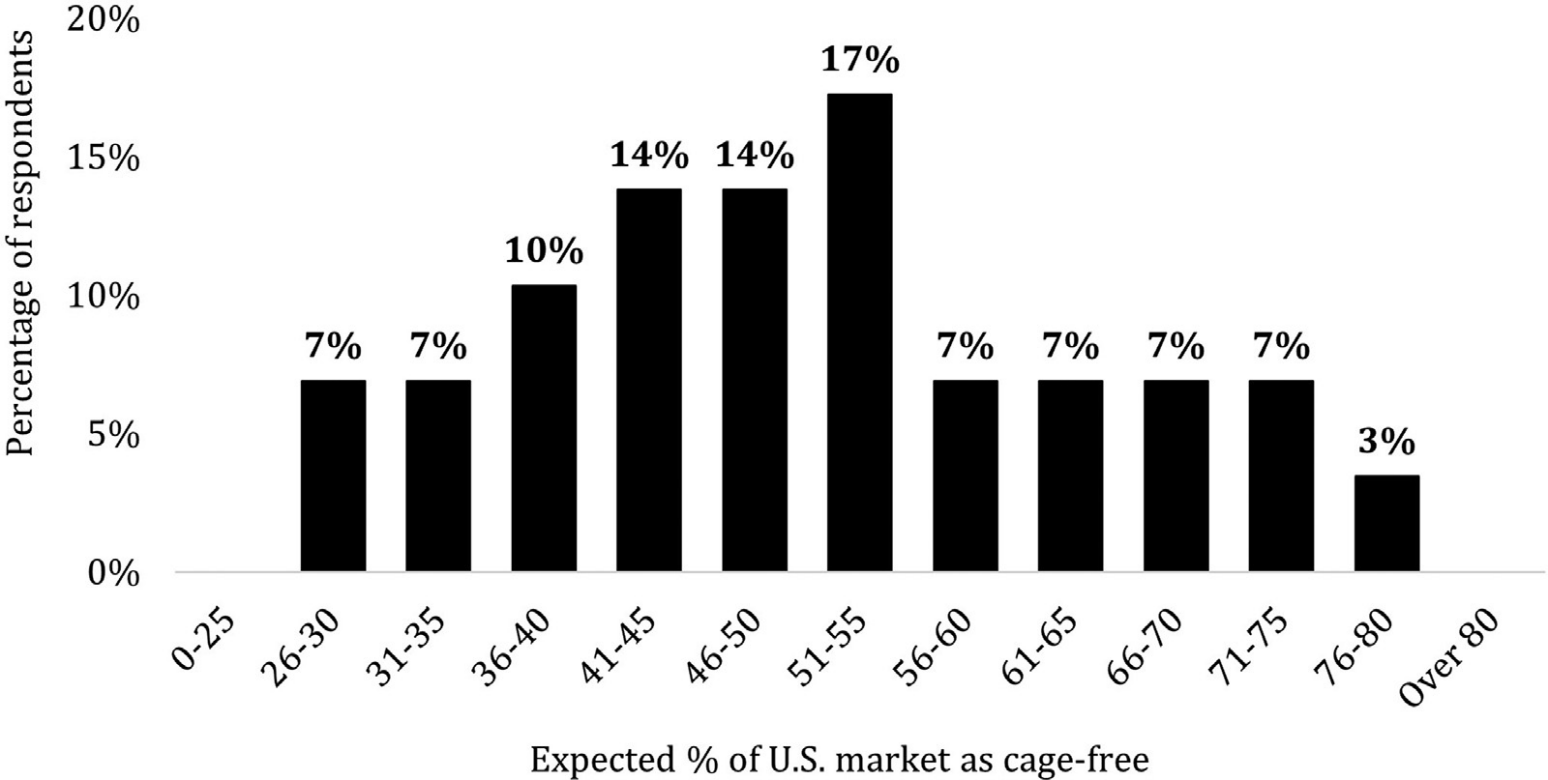
Views on national cage-free market share, January 2026.

Knowing that 75% of the egg industry would need to convert by January 2026 to cage-free to meet retailer pledges (Lusk, 2019), we asked producers what percentage of the industry they expect to be cage-free by this time. Figure 6 offers a distributional summary of responses. On average, respondents believe that 29% of U.S. laying hens are currently housed in cage-free systems. By January 2026, respondents expect 51% of the industry to be cage-free; just 10% of producers believe cage-free production will reach the three-quarter threshold by this time. Thus, producers are skeptical about the feasibility that retailer pledges can be met by 2026. This skepticism is unsurprising, as producers also view cage-free as more of a challenge than an opportunity.

Producers were asked on a 5-point scale (spanning from “enormous opportunity” to “enormous challenge”) what best aligns with their view for the national industry regarding a possible increase in cage-free production. No producer responded “enormous opportunity,” 5 (18%) responded “opportunity,” 10 (36%) responded “equally balanced opportunity & challenge,” 5 (18%) responded “challenge,” and 8 (29%) responded “enormous challenge.”

## DISCUSSION AND IMPLICATIONS

Given the pressure from animal advocacy organizations (Lusk and Norwood, 2011), the challenges of complying with different state regulations (Staples et al., 2022), and a desire to protect brand image (Kapelko and Oude Lansink, 2022), many food retailers have pledged to fully convert to cage-free by January 2026. While the share of eggs produced in cage-free systems has risen steadily over the past decade, the rate of increase is insufficient to meet all existing pledges by the January 2026 deadline. Moreover, final retail egg consumers are not choosing to buy cage-free eggs over conventional at a rate where retailer pledges would be automatically fulfilled by the market alone. As such, this mixed-methods study was designed to understand why retailer pledges have yet to be fulfilled and identify what changes might alleviate the conundrum.

According to the producers in our study, higher costs are the main challenges in further transitioning to cage-free housing. These costs include greater labor, feed, and capital requirements. Much of this sentiment aligns with previous studies on producer responses to agricultural policies restricting farming practices (Tuyttens et al., 2011; Matthews and Sumner, 2015; Stadig et al., 2016). These studies suggest that conventional cages are financially superior in fixed and operating costs, affecting the farm's overall profitability potential. For example, Matthews and Sumner (2015) suggest that operating costs in cage-free systems are 23% higher than in conventional systems, primarily driven by the differences in labor and feed costs. Additionally, the authors indicate that average total costs in cage-free systems are 36% higher than conventional systems due to the higher fixed capital costs, lower stocking densities, etc.

With higher production costs, egg producers state that food affordability and insecurity is one barrier preventing further cage-free adoption. Indeed, it is well-documented that implementing more restrictive on-farm practices increases consumer costs (Malone and Lusk, 2016; Mullally and Lusk, 2018; Carter et al., 2021; Oh and Vukina, 2021), as heightened production costs are often pushed downstream onto the consumer. Intuitively, if the conventional alternative is removed from the market, the supply curve shifts to the left and increases the average cost per dozen eggs. This results in a decrease in equilibrium quantity, meaning fewer eggs are bought and sold in the market, where the magnitude of the reduction will depend on the percentage change in price and slope of the demand curve (i.e., the own-price elasticity of demand for eggs). Furthermore, the price increase could be most detrimental to the lowest income bracket that relies on eggs as a cheap source of protein (Sumner et al., 2011).

Egg producers also raise caution over the environmental impact of cage-free systems relative to conventional systems. In the literature, Leinonen et al. (2012) and Shepherd et al. (2015) support this concern, suggesting that the increased hen activity and lower stocking densities of cage-free systems lead to higher environmental impacts relative to conventional systems.^13^

The final concern among producers was the extent to which the transition toward cage-free production might favor large vs. small producers, which might increase consolidation alongside the transition to cage-free production. A bank's decision to approve a loan will depend on the value of the firm's assets, and as cages lose their value, it could become more difficult for firms to secure funding (as stated by Producer D). This is particularly true for smaller firms with a weaker market position, and this precautionary lending could raise concerns over industry consolidation. For example, Stadig et al. (2016) provide evidence of EU firms exiting the egg market following the ban on conventional systems. One-third of the producers in their sample left the market following the policy change, with 72% attributing their departure to the more restrictive practice. As the current U.S. administration has expressed concern over industry consolidation in protein markets (e.g., Executive Order 14036, 2021), the transition to cage-free could have unintended consequences related to industry consolidation.

The voluntary nature of the cage-free commitments and the market structure of the U.S. egg industry makes the U.S. transition unique to the EU cage-free transition outlined in Stadig et al. (2016) and Tuyttens et al. (2011). While 10 states have passed legislation to phase out conventional eggs, most of the transition relies on the soft commitments made by retailers. This adds a layer of complexity to the U.S. cage-free egg market transition, heightening risk and uncertainty in investment decisions. With the challenges associated with the cage-free transition, egg producers are skeptical that the industry can meet all existing pledges by 2026. These challenges highlight the need to understand the potential ramifications of the cage-free transition across the supply chain. Understanding these aspects is critical for producers and processors to make informed production decisions and long-term investments. Extending the pledge deadlines could give egg producers more time to acquire financing from lenders and better coordinate the depreciation of current housing stock with the construction of new facilities.

From a marketing and policy perspective, the data offer some insights into ways to facilitate the conversion to cage-free housing. To the extent there are concerns about inequalities or consolidation, retailers could offer differential terms, including extended timelines that vary by the size of the producer. Offering cost-plus pricing contracts will likely be most attractive to egg producers in encouraging a transition. While subsidies to incentivize the conversion to cage-free production could be an option, producers were generally against government-backed support. This sentiment likely stems from the fact that subsidies to fund construction only solve the short-run capital problem and cannot solve the long-term problem of securing a buyer in a higher-cost system. Ultimately, egg producers are sensitive to the needs of retailers and highlighted the fact that retail demand would ultimately drive the transition to cage-free (or not).^14^

Of course, it is also impossible to view cage-free mandates and pledges in a vacuum. Egg prices reached record highs in January 2023 (BLS, 2023), driven by avian influenza, supply chain disruptions, etc. (Muhammad et al., 2023). In the wake of current macroeconomic conditions and supply chain constraints, producers believe the timeline for the transition to cage-free must better reflect current market conditions, equipment constraints, and other logistical challenges.

The primary limitation of this study is the sample size: 7 qualitative interviews and 29 quantitative responses. Despite this apparent small sample, the results represent the perspectives of the Presidents and CEOs of a nontrivial share of the U.S. market. The 7 qualitative interviews collectively account for more than 25% of the U.S. egg industry, while the 29 quantitative responses account for 40 to 50% of the market. For comparison, the 140 responses from Belgian egg producers presented in Tuyttens et al. (2011) represent only 0.01% of the EU market (Windhorst, 2017).^15^ Thus, our observations represent a large share of the U.S. egg market given the industry's production landscape. We hope that the results can offer important insights into the impending transition to cage-free production and the potential unintended consequences of policy mandates and retailer pledges.

There remain several important avenues for future research. For instance, in the qualitative portion of this study, producers express their perceptions of the future of the industry in the form of barriers and opportunities. Future work could concentrate on a single metric (e.g., food affordability and insecurity) through a quantitative lens and identify how the cage-free transition will affect egg prices and the purchasing behavior of different income groups. A second area for future work is tracking individual producer decision-making over time, similar to the approach of Stadig et al. (2016) in Belgium. This would involve a longitudinal survey to understand how investment decisions, contracting, etc., evolve as the transition continues. Finally, future studies should consider the attitudes, expectations, and decision-making of other stakeholders across the egg supply chain. While researchers have analyzed consumer behavior and sentiment toward cage-free eggs (e.g., Doyon et al., 2016; Lusk, 2019; Cao et al., 2021), less is known about retailers’ adjustment to cage-free markets. A study analyzing the retailer perspective could link what is already known about the start- and end-points of the egg supply chain (i.e., producers and consumers) and fill an essential gap in the literature.

## CONCLUSIONS

Ten states and many food retailers have pledged to phase out the sale of conventional eggs by January 2026. Yet uncertainty remains about whether egg producers can transition at a rate proportionate to demand. This mixed-methods paper analyzes producer opinions, expectations, and sentiment surrounding the transition to cage-free production using data from qualitative and quantitative surveys. Across both studies, producers described the differences between the conventional and cage-free production systems and echoed common barriers preventing the transition. Higher fixed and variable costs, limited customer demand, and higher environmental impact were among the most notable challenges to cage-free adoption. With heightened production costs, lower production efficiency, and increased production risk, there is also concern over food affordability and food security. Ultimately, producers are highly skeptical that the industry can reach the cage-free target necessary to meet retailer pledge deadlines. As the country pivots toward a higher cost, lower efficiency system with a higher environmental footprint, producers emphasize caution in implementing mandates and pledges. They also encourage transparency and honest conversations about the effects of cage-free mandates and pledges on the egg supply chain with their customers, policymakers, and consumers.

## DISCLOSURES

This research was partially funded by the United Egg Producers and Food Industry Association—FMI.

## Footnotes

1 It is critical to note here and throughout this manuscript that these 7 producers represent more than 30% of the U.S. egg market. Similarly, the 29 producers that responded to our quantitative producer survey (Study 2, presented later) represent more than 30% market share. This implies that despite the small samples, and given the industry's structure, our results represent the perspectives of a large share of the industry. Other studies that have evaluated producer decision-making have larger sample sizes but represent a smaller share of the market (e.g., Tuyttens et al., 2011; Tonsor and Schulz, 2020). For comparison, the study that most closely resembles ours is Tuyttens et al. (2011). While they have 140 responses from Belgian egg producers, their aggregate production accounts for less than 1% of the EU market. Thus, we believe that our results can help inform the transition to cage-free even with the small sample size.

2 This coincides with a report from Alltech (2022) suggesting a 7 to 8% increase in feed intake for cage-free flocks compared to conventional ones.

3 Mortality costs across systems is also debated in the literature. For example, while Weeks et al. (2016) state that mortality rates are higher in cage-free systems, research from Freire and Cowling (2013) and Schuck-Paim et al. (2021) support a declining cage-free mortality rate as producers gain more experience.

4 The anticipated share of eggs ending as floor eggs differed across producers with some of our interviewees reporting losses up to 20% floor eggs at times (Producer D), while others indicated a share of around 5-6% (Producer A). Nevertheless, producers agreed that achieving 1% floor eggs was the goal, though a 1% loss still represents a major loss.

5 Three producers explicitly described some of their contractual agreements as need-based, where the contract does not specify a specific quantity but rather an agreement to meet the need of the customer; 2 of 7 producers describe having informal handshake agreements with buyers.

6 To this point, 3 of the 7 producers used the phrase “consumers vote with their wallet” to describe the disconnect between stated policy preferences and revealed purchasing behavior.

7 When discussing the limitations to entrepreneurship and innovation on the production side, Producer B stated, “I think any entrepreneurship is going to get mowed down by the scale of production.” Producer C added that an entrepreneur can “start small and try to build a niche brand, but by the time you look at the land and all the other resources… the capital requirements are so enormous. It's such a barrier.”

8 Even among large producers, the excitement over cage-free was limited (except for Producer G, who has already transitioned more than 60% of their facilities to cage-free). For example, Producer E stated, “there is an opportunity for additional revenue, but it also comes at a cost. So ultimately, it could cancel out.”

9 It is worth noting how the 2 studies were designed to understand the link between them. Before data collection, we planned a mixed-methods approach such that statistical insights could be supported by producer anecdotes and vice versa. In doing so, we constructed the studies around the same four themes: i) current business operations, ii) comparison of production systems, iii) business plan and relationship with buyers, and iv) future industry directions. After deciding on these themes, we created the survey instrument for the qualitative semistructured producer interviews. These questions were then used to create an initial draft of the quantitative study. Adjustments were made where appropriate to create a user-friendly survey (for both the survey participant and researchers) and in accordance with standard field practices. For example, when asking the quantitative survey participants about their current operation size, they choose between a multiple-choice question with different annual production ranges; rather than an open-ended question where they type in their response. The qualitative interviews then occurred in June 2022. These data were analyzed using qualitative content analysis, allowing us to identify common trends in producer responses. Additional questions were then added to the qualitative study, and some were removed, to emphasize or de-emphasize topics commonly discussed in the qualitative portion of the project. Thus, the 2 studies were linked in common themes, but conducting the qualitative interviews first allowed the quantitative study to focus on the most pertinent information.

10 A total of 68 producers began the survey. To demonstrate when the other 39 respondents exited the survey, 48 answered the first question, 38 answered the fifth question, 34 answered the 10th question, and 30 answered the 24th question. Complete documentation of survey responses received is in the supplemental material accompanying this text. To assess the possible impacts of focusing on the 29 fully complete surveys, we compare feedback from the fully available sample with the complete surveys. This comparison indicates that both samples are very similar in their assessment of current cage-free prevalence in the United States and expectations for the number of laying hens in January 2026 that may produce table or market-type eggs. Combined, this adds some reassurance regarding focusing on the 29 complete surveys.

11 Cage-free annual costs being 18% higher for labor, 11% higher for feed, 17% higher for fixed/non-operating capital, 16% higher for variable/operating capital, 9% higher for electrical/utilities, 8% higher for repairs/maintenance, 11% higher for mortality, and 8% higher for morbidity when compared to conventional housing. We also ask respondents to compare the annual costs across categories when transforming an existing facility to cage-free. This leads to average estimates that are comparable to those presented here and described in the main text.

12 The revenue calculation reflects an interval-censored model presuming lower-end values of −10% (3 over 5% lower than conventional responses) and higher-end values of +25% (4 over 20% higher than conventional responses). The ROI calculation reflects interval-censored models presuming lower-end values of −4% (1 cage-free response of below 1%) and higher-end values of +25% (2 cage-free and 1 conventional response of over 20%).

13 Leinonen et al. (2012) use a life-cycle assessment to explore the environmental impacts and global warming potential of different egg production systems. Their results indicate that the conventional system requires less feed, water, and housing or land than cage-free systems. With fewer input requirements, the authors also suggest lower eutrophication and acidification potential, which are commonly used metrics for assessing environmental concerns in agriculture systems. They suggest that the cage system also has lower electricity use for ventilation, lighting, etc., which contributes to the lower primary energy use and global warming potential. Shepherd et al. (2015) also suggest that cage-free systems have higher carbon dioxide emission rates than cage systems, likely driven by increased hen activity and lower stocking densities.

14 For example, Producer F claimed, “We don't have a preference on the style of production, cage-free versus conventional. But our models are predicated on understanding the customer and delivering what they want, where they want it.”

15 Tuyttens et al. (2011) present an average flock size of 32,160 laying hens amongst their 140 respondents. Compared to the number of laying hens in Belgium in 2010 (9.2 million; Windhorst, 2017), the data from Tuyttens et al. (2011) represent approximately 48% of Belgian laying hens. However, when compared to the relative number of EU laying hens in 2010 (362.6 million; Windhorst, 2017), it represents less than 1% of the EU market.
